# CARING FOR CAREGIVERS: HEALTH AND HEALTHCARE EXPERIENCES OF DIRECT CARE WORKERS

**DOI:** 10.1093/geroni/igac059.625

**Published:** 2022-12-20

**Authors:** Stephen McCall

**Affiliations:** Altarum, Saint Petersburg, Florida, United States

## Abstract

Direct care workers play a significant role in supporting the health of older adults and people with disabilities across long-term care settings—but as a primarily low-income, women of color workforce, they likely face significant health risks themselves. To build understanding on the health of this essential workforce, this paper examines direct care workers’ health status, health insurance coverage, and experiences utilizing, accessing, and paying for healthcare services using pooled 2014 to 2018 data from the National Health Interview Survey. We find direct care workers have worse health, lower rates of insurance coverage, poorer access and utilization, and greater cost barriers compared to other healthcare workers. We also observe disparities within the direct care workforce according to industry, gender, and race/ethnicity. This study helps inform efforts to support the health of direct care workers and stabilize the critical services they provide.

